# Dataset of energetics and biomechanics of self-paced and fixed speed treadmill walking at multiple speeds

**DOI:** 10.1016/j.dib.2022.107915

**Published:** 2022-02-08

**Authors:** Kyra Theunissen, Bas Van Hooren, Guy Plasqui, Kenneth Meijer

**Affiliations:** a Department of Nutrition and Movement Sciences, School of Nutrition and Translational Research in Metabolism, Maastricht University Medical Centre, Minderbroedersberg 4-6, Maastricht 6211 LK, The Netherlands; b Rehabilitation Research Center, REVAL, Faculty of Rehabilitation Sciences, Hasselt University, Belgium; c School of Care and Public Health Research Institute, Maastricht University Medical Centre, The Netherlands

**Keywords:** Gait, Biomechanics, Energy cost of walking, Self-paced treadmill, Energy expenditure, Kinematics

## Abstract

**Data collection process:**

This dataset includes continuously measured walking biomechanics collected using three-dimensional motion capture and gas exchanges of 18 healthy participants (9 male/9 female, mean ± standard deviation age 24.8 ± 3.3 years, height 1.71 ± 0.81 meter, weight 65.9 ± 8.1 kilogram). Walking biomechanics were recorded during four different self-paced speeds (comfortable, very slow, slow, fast) in randomized order and four fixed-paced speeds on an instrumented treadmill. The average walking speed during the last two minutes of a 6 minute self-paced walking familiarization period was determined as the comfortable walking speed and used to set the target speed ranges for the other self-paced conditions: slow (20% slower than comfortable), very slow (40% slower) and fast (20% faster) Oxygen consumption (O_2_) and carbon dioxide (CO_2_) production were measured continuously and computed at 5-second intervals throughout the resting metabolic rate (RMR) measurement and walking trials. RMR (J·24 hours) was computed from the average O_2_ and CO_2_ measured during the last 5 minutes of the 35 minutes RMR measurement using Weir's non-protein equation. The energy consumption of walking (J·min^−1^) at each speed was computed from the average O_2_ and CO_2_ measured during the last 2 minutes of each condition using similar procedures. RMR (J·min^−1^) was subtracted from the energy consumption of walking to determine net walking energy consumption. The net cost of walking was then expressed as J·kg·0.67^−1^·m^−1^. All participants avoided strenuous activity 24 hours, and eating and drinking (with the exception of water) up to 3hours before the session. Height was measured using a stadiometer (SECA, model 213, Hamburg, Germany). Body mass was measured by force platforms.

**Analysis performed:**

Both the exported data files from the CAREN software (D-flow) (.mox files), and processed data files with a custom-made Matlab script are included. Marker and force plate data in the .mox and processed files were low-pass filtered using a 2^nd^ order Butterworth with a cut-off frequency of 6 Hz. C3D files with raw marker and ground reaction force data are available upon request.

**Data:**

Continuously measured spatiotemporal parameters, energetics, 3D lower lumb plus trunk kinematics, 3D kinetics and surface muscle activation during walking at both self-paced and imposed(fixed) speeds on a treadmill. Resting metabolism.

**Reuse potential:**

1) Assessing self-paced and fixed speed treadmill walking biomechanical and energetics, 2) assessing biomechanics and energy expenditure at multiple or particular speeds, 3) investigating the relationship between walking biomechanics and energetics, 4) reference database of walking biomechanics and energetics of healthy adults.

## Specifications Table

**Table:** 

Subject	Pulmonary and Respiratory Medicine Walking biomechanics
Specific subject area	Energetics, biomechanics of treadmill walking at a fixed and feedback controlled (self-paced) speed
Type of data	Excel files with energetics, kinetics, kinematics during walking. SPSS files with all data, .mox files with marker coordinates, ground reaction forces, and surface muscle activation. C3D files with raw marker and ground reaction force data is available upon request.
How data were acquired	The computer assisted rehabilitation environment (CAREN, Motek, The Netherlands) system combines an instrumented split-belt treadmill (ForceLink, Culemborg, The Netherlands, individual belt length and width 2.15 × 0.5 m, 6.28-kW motor per belt, 60 Hz belt speed update frequency, and 0-18 km·h^−1^ speed range, 1000 Hz) with a 12-camera three- dimensional motion capture system (VICON NEXUS v2.7, Oxford Metrics Group, Oxford, UK, 100 Hz) and was used to measure kinetic and kinematic outcomes during. Respiratory gases were captured using a total- capture indirect calorimeter (Omnical, Maastricht Instruments, Maastricht The Netherlands)
Data format	Raw Analyzed Filtered
Parameters for data collection	Individuals that were comfortable with treadmill walking, aged 18-35, had a body mass index (BMI) of <30, free of injuries or diseases that could affect gait and could walk continuously for a minimum of 40 minutes were recruited. All participants completed a single test session and were instructed to avoid strenuous activity 24 hours, and eating and drinking (with exception of water) up to 3hours before the session. All subjects wore standardized shoes to decrease the contribution of insoles/shoe cushioning to walking biomechanics/or: to reflect barefoot walking mechanics and were attached to an overhead suspension system to prevent falls in case a subject lost balance.
Description of data collection	When entering the lab, Resting Metabolic Rate (RMR) was measured via indirect calorimetry while participants lay down for 35 minutes in supine position. Afterwards participants walked for six minutes in self-paced mode as familiarization with treadmill walking and wearing of the face mask. The average walking speed during the following two minutes was determined as the comfortable walking speed and used to set the target speed ranges for the other randomized self-paced conditions: slow, very slow and fast with 20% and 40% slower and 20% faster than comfortable, respectively. All conditions were performed for 4 minutes, with data collection being started in the last 2 minutes to ensure a steady-state in oxygen consumption and wash-out of former speeds. The average walking speed during the last 2 minutes of the self-paced trials was used to match the speed in fixed-paced conditions. During all self-paced trials, the participants were provided with real-time feedback about their walking speed using the virtual environment projected onto a 180 degrees screen and instructed to find a comfortable pace within a ±5% range of the target speed. In the self-paced condition, the belt speed was adjusted based on the position of the participant (determined using the pelvis markers) relative to the middle of the belt and the speed of the participant. For example, the belt speed increased when the participant was walking in the front of the treadmill, with the acceleration increasing as the participant walked more towards the front of the treadmill. The sensitivity of the self-paced algorithm was set to default (i.e., 1) as this has been shown to best match self-paced overground walking variability in healthy individuals.
Data source location	Institution: Maastricht University Medical Centre^+^ City/Town/Region: Maastricht Country: Netherlands
	Please consult the article for speciation of method. Repository name: OSF.io Data identification number: [provide number, if available] Direct URL to data: [e.g. https://doi.org/10.17605/OSF.IO/AENRW
Related research article	Theunissen, K., Van Hooren, B., Plasqui, G., Meijer, K., 2022. Self-paced and fixed speed treadmill walking yield similar energetics and biomechanics across different speeds. Gait & Posture 92, 2–7. doi:10.1016/j.gaitpost.2021.11.005

## Value of the Data

• The consecutive and continuous measurements of this data can improve understanding of energetics and biomechanics of treadmill walking at different speeds at both self-paced and fixed paced speeds.
• The data can improve understanding differences between self-paced and fixed paced walking.
• The data can improve understanding of the energy cost of walking at different speeds and the corresponding biomechanics in healthy individuals.
• The data can be used as part of a reference database for walking biomechanics and energetics.
• The range in very slow to fast speeds can be used for different populations.
• The data can be used to investigate associations between walking biomechanics and energetics at different speeds.
• The data can be useful for any scientist interested in gait.
• The data can be used for any scientific purposes in case permission is granted.

## 1 Data Description

Can be found in the file open access file information_OSF.

**Table:** 

**Coding and structure**					
Folder	File type	Content	Generated by	Naming	Additional
general OSF storage folder	.sav	SPSS dataset outcome measures	SPSS (combinati on of .xlsx files)	Self_fix_SPSS_wide _OSF	/
general OSF storage folder	.pdf	Technical Information for interpreting .xslx processed gait data	User	Technical form_CAREN.pdf	/
gait data .mox files	.mo x	ID data, marker data, gait data	D-flow (Computer Assisted Rehabilitati on Environment (CAREN) system)	Study name_participant code_condition_speed	/
processe d gait data .xlsx	.xls x	Spatiotemporal parameters, Kinematics, Kinetics	Private matlab script for analyzing .mox data	Study and/or participant code_condition_sp eed	Consider ``Technical form_CAREN.pdf'' for interpreting the data
energy expenditure	.xls x	Energy expenditure During resting and walking	Omnical Indirect calorimeter	Study and/or participant code_condition_sp eed Study and/or participant code_BMR or RMR: resting metabolism	SF 1-10 different file per speed SF11-18 all speeds in one file

## 2 Experimental Design, Materials and Methods

The computer assisted rehabilitation environment (CAREN, Motek, The Netherlands) system combines an instrumented split-belt treadmill (ForceLink, Culemborg, The Netherlands, individual belt length and width 2.15 × 0.5 m, 6.28-kW motor per belt, 60 Hz belt speed update frequency, and 0-18 km·h-1 speed range, 1000 Hz) with a 12-camera three-dimensional motion capture system (VICON NEXUS v2.7, Oxford Metrics Group, Oxford, UK, 100 Hz) and was used to measure kinetic and kinematic outcomes during walking. The force platform was zeroed, and the cameras were calibrated with the dynamic calibration procedure embedded in Vicon prior to measurements. In line with the International Society of Biomechanics (ISB) guidelines, each participant was asked to perform basic motion tasks of the hip and knee joint to determine the “functional” axis of rotation and joint center. Five flexion-extension movements per leg were performed for the knee, and one star arc movement was performed per leg to determine the “functional” knee and hip joint axis of rotation, respectively. Respiratory gases were captured using a total-capture indirect calorimeter (Omnical, Maastricht Instruments, Maastricht The Netherlands). The participants wore a face mark (Hans Rudolp Inc, Shawnee, KS, USA) over the nose and mouth and care was taken to use a mask that fitted without detectable leakage. The mask was connected to a T-piece that was placed in a free airstream that was passing at 200 L min-1 (during walking) or 100 L min-1 (for resting metabolism) through the tubes. The system was calibrated automatically every 15-30 minutes using room air and a gas mixture of known composition. Nitrogen gas (>99.999%, Linde, Schiedam, The Netherlands) was used to set the zero and a calibration gas with 18% O_2_ and 0.8% CO_2_ certified to 1% volumetric content was used for calibration (HiQ specialty gas, Linde).

## Ethics Statement

The study was approved by the local ethics committee (nr. 2019-1128), was conducted in compliance with the declaration of Helsinki and all participants signed informed consent form prior to the measurements.

## CRediT Author Statement

**Kyra Theunissen and Bas van Hooren:** Data collection; **Kenneth Meijer and Guy Plasqui:** supervision. All authors contributed to the conceptualization, methodology, formal analysis, writing – reviewing & editing the manuscript and approved the final version prior to submission.

## Declaration of Competing Interest

KT was funded by special research fund 2017 call for doctoral grants in the framework of BOF UHasselt

– Maastricht UMC+ cooperation.

BVH was funded by the Kootstra Talent Fellowship awarded by the Centre for Research Innovation, Support and Policy (CRISP) of Maastricht University Medical Center+.

The authors declare that they have no known competing financial interests or personal relationships which have or could be perceived to have influenced the work reported in this article.

